# Enhancing ASPP2 promotes acute liver injury via an inflammatory immunoregulatory mechanism

**DOI:** 10.3389/fimmu.2024.1381735

**Published:** 2024-05-22

**Authors:** Xiangying Zhang, Ling Xu, Zihao Fan, Yao Gao, Yuan Tian, Yaling Cao, Dexi Chen, Feng Ren

**Affiliations:** Beijing Institute of Hepatology/Beijing Youan Hospital, Capital Medical University, Beijing, China

**Keywords:** acute liver injury, autophagy, inflammation, apoptosis, ASPP2

## Abstract

**Background:**

Acute liver injury (ALI), which is a type of inflammation-mediated hepatocellular injury, is a clinical syndrome that results from hepatocellular apoptosis and hemorrhagic necrosis. Apoptosis stimulating protein of p53-2 (ASPP2) is a proapoptotic member of the p53 binding protein family. However, the role of ASPP2 in the pathogenesis of ALI and its regulatory mechanisms remain unclear.

**Methods:**

The expression of ASPP2 were compared between liver biopsies derived from patients with CHB, patients with ALI, and normal controls. Acute liver injury was modelled in mice by administration of D-GalN/LPS. Liver injury was demonstrated by serum transaminases and histological assessment of liver sections. ASPP2-knockdown mice (ASPP2^+/−^) were used to determine its role in acute liver injury. Mouse bone marrow macrophages (BMMs) were isolated from wildtype and ASPP2^+/-^ mice and stimulated with LPS, and the supernatant was collected to incubate with the primary hepatocytes. Quantitative real-time PCR and western blot were used to analyze the expression level of target.

**Results:**

The expression of ASPP2 was significantly upregulated in the liver tissue of ALI patients and acute liver injury mice. ASPP2^+/-^ mice significantly relieved liver injury through reducing liver inflammation and decreasing hepatocyte apoptosis. Moreover, the conditioned medium (CM) of ASPP2^+/-^ bone marrow-derived macrophages (BMMs) protected hepatocytes against apoptosis. Mechanistically, we revealed that ASPP2 deficiency in BMMs specifically upregulated IL-6 through autophagy activation, which decreased the level of TNF-α to reduce hepatocytes apoptosis. Furthermore, up-regulation of ASPP2 sensitizes hepatocytes to TNF-α-induced apoptosis.

**Conclusion:**

Our novel findings show the critical role of ASPP2 in inflammatory immunoregulatory mechanism of ALI and provide a rationale to target ASPP2 as a refined therapeutic strategy to ameliorate acute liver injury.

## 1 Introduction

The global burden of liver disease is tremendous, and acute liver injury (ALI) is especially associated with high mortality and poor prognosis (1). ALI frequently results from viral hepatitis, ingestion of drugs or toxic substances, or hepatic ischemia-reperfusion injury. Unfortunately, there is currently no effective therapy for the end stage of the disease other than liver transplantation because the core mechanisms of ALI are still not well understood (2). Therefore, understanding the molecular mechanisms that contribute to the onset and progression of ALI is urgently needed.

Liver inflammation, especially cytokine storms, plays a critical role in the pathophysiology of ALI and mediates liver injuries that are closely related to hepatocyte proliferation and regeneration (3, 4). During the progression of ALI, Kupffer cells initiate transduction and amplification of the “alarm” signal following an injurious event; in addition, hepatocyte death leads to the release of damage-associated molecular patterns (DAMPs), which activate innate immune and tissue destructive responses through the production of inflammatory mediators, chemokines, and reactive nitrogen and oxygen species (5). Subsequently, hepatic and circulating inflammatory cytokines form a positive feedback loop in the innate immune system; the overproduction of cytokines is hazardous to the host and may cause severe outcomes. Therefore, strategies that attenuate the initiation of the inflammatory response and mitigate the deleterious systemic effects of proinflammatory cytokine secretion should be considered.

ASPP (ankyrin-repeat-containing, SH3-domain-containing, and proline-rich region-containing protein) family members, including ASPP1, ASPP2, and iASPP, are apoptosis regulatory proteins. ASPP1 and ASPP2 enhance, whereas iASPP inhibits, the activities of p53 and its family members p63 and p73. ASPP2 is a haploinsufficient tumor suppressor, and aberrant expression of ASPP2 has been found in a variety of human cancers, including liver cancer, lung cancer, breast cancer, and leukemia, and this factor has been implicated in cancer development and progression (6, 7). In nontumor diseases, recent studies have shown that ASPP2 attenuates triglycerides to protect against hepatocyte injury by reducing autophagy in cell and mouse models of nonalcoholic fatty liver disease (8), and ASPP2 inhibits the profibrotic effects of transforming growth factor-β1 in hepatic stellate cells by reducing autophagy (9). Furthermore, using computational methods to study the interaction between nuclear factor-κB (NF-κB) and ASPP2, Assaf Friedler et al. demonstrated that ASPP2-mediated inhibition of NF-κB was a possible mechanism by which ASPP2 exerts its apoptotic effect (10). The study also showed that ASPP2 is a transcriptional target of STAT1 in response to LPS and IFN signaling and revealed an important function of ASPP2 in the response of cells to infection and inflammation (11). However, it remains unknown whether ASPP2 is involved in the pathogenesis of ALI and its regulatory mechanisms.

The present study was conducted to test our hypothesis that the expression of ASPP2 is upregulated in the liver during ALI and promotes the inflammatory response to promote liver injury. The results revealed that elevated ASPP2 expression was observed in the livers of human ALI patients and ALI mouse models, and knockdown of ASPP2 significantly ameliorated liver inflammation and decreased hepatocyte apoptosis. Furthermore, we demonstrated that ASPP2 in bone marrow-derived macrophages (BMMs) specifically downregulated IL-6 and increased TNF-α through autophagy inhibition.

## 2 Materials and methods

### 2.1 Human specimens

10 normal liver tissue samples were collected from 4 subjects who underwent hepatic resection for hepatic cysts, 3 subjects who underwent hepatic resection for colorectal metastasis, and 3 subjects who underwent hepatic resection for benign tumors. 14 samples of chronic hepatitis B (CHB) were obtained from patients who underwent liver puncture biopsy, and 19 samples of acute liver failure (ALI) were obtained from patients with HBV-related ALI who underwent liver transplantation. The clinical characteristics and details of all subjects are described in **Supplementary Table 1**. This study adheres to the ethical principles of the 1975 Declaration of Helsinki and has been approved by the Medical Ethics Committee of Beijing YouAn Hospital. Informed consent was obtained from all patients.

### 2.2 Animal experiments

Wild-type or ASPP2^+/-^ BALB/c male mice were used in this study. The SPF grade BALB/c mice, 12∼16 weeks old, were purchased from Jiangsu Jicui Pharmachem Biotechnology Co. Ltd. (Production License No.: SYXK 2018-0008). The ASPP2^+/-^ mice were prepared with the CRISPR/Cas9 system, the sgRNA oligonucleotides were constructed to target ASPP2, and this work has been published (12). Briefly, a mixture of sgRNA and cas9 was microinjected into the fertilized eggs of BALB/c mice using a microinjector to cleave introns 1-2 and 3-4 of ASPP2, and then transplanted into the uteri of pseudopregnant BALB/c females to await the birth of F0 generation mice. At 5-7 days after birth of the F0 mice, the tail tissue was excised and DNA was extracted, and the primers designed in the target region were used for PCR identification, and the PCR-positive samples were selected for sequencing. The selected F0 generation mice were mated with wild-type mice to produce F1 generation mice; the F1 generation mice were also identified by PCR and sequencing, and the resulting positive heterozygous F1 generation mice could be stably inherited.

The mice were intraperitoneally injected with LPS (10 μg/kg; InvivoGen, San Diego, CA, USA) 15 minutes before D-GalN (700 mg/kg; Sigma, St. Louis, MO, USA) to induce ALI. Suppression of autophagy was achieved by tail vein injection of small interfering RNA (siRNA) for autophagy gene 7 (Atg-7, 3 mg/kg). Control mice received the same volume of saline. The mice were sacrificed at various time points after D-GalN/LPS treatment, and liver and serum samples were collected for further analysis. All the experimental methods performed in the animal studies were approved by the Ethical Committee of Capital Medical University.

### 2.3 Serum aminotransferase activity

Whole blood was collected from the mice 6 h after D-GalN/LPS administration, which centrifugated at 3000 x g for 15 minutes to acquire serum samples. Serum levels of alanine aminotransferase (ALT) and aspartate aminotransferase (AST) were measured by using a multiparametric analyzer (AU 5400, Olympus, Japan) according to the manufacturer’s instructions.

### 2.4 Histopathological analysis

Liver tissues were fixed in 4% paraformaldehyde and embedded in paraffin wax, and then 5 µm sections were sliced. These sections were stained with hematoxylin and eosin (H&E) using a standard protocol and then analyzed by light microscopy.

### 2.5 TUNEL assay

Hepatocyte apoptosis in liver sections was detected by terminal deoxynucleotidyl transferase dUTP nick-end labeling (TUNEL, red fluorescence) using an *In Situ* Cell Death Detection Kit (Roche, Indianapolis, IN). A negative control was prepared through the omission of terminal transferase. Positive controls were generated by treatment with DNase. Liver sections were permeabilized in 0.01% Triton X-100 (Sigma, Saint Louis, MO) for 20 min at room temperature, and then incubated with TUNEL working solution for 1 h at 37° C in the dark. Then, nuclei were stained with 4,6-diamino-2-phenylindole (DAPI, 1 μg/ml) for 10 min. Images were obtained on a Nikon Eclipse E800 fluorescence microscope.

### 2.6 Quantitative real-time PCR analysis

Total RNA was isolated from liver samples using TRIzol reagent according to the manufacturer’s protocol. To be detailed, add 1 mL of TRIzol™ Reagent per 50–100 mg of tissue to the sample and homogenize using a homogenizer and then incubated for 5 minutes at room temperature; 0.2 mL of chloroform were added in the lysis and thoroughly mix by shaking; incubate for 2 minutes and then centrifuge the sample for 15 minutes at 12,000 × g at 4°C; finally, Transfer the aqueous phase containing the RNA to a new tube.

A total of 2.5 μg of RNA was reverse-transcribed into cDNA using the SuperScript™ III First-Strand Synthesis System (Invitrogen, Carlsbad, CA, USA). To be detailed, total RNA were mixed with 50ng random hexamers, 10mM dNTP mix and DEPC-treated water up to 10μl; the mixture were incubated at 65°C for 5 min; and then mixed the product with: 10x RT buffer (2μl), 25mM MgCl_2_ (4μl), 0.1M DTT(2μl), RNase(1μl) and SuperScript^®^ III RT(1μl) to obtain cDNA Synthesis Mix (20μl). The mix was incubate as follows: 10 min at 25°C, followed by 50 min at 50°C, 85°C for 5 min and chill on ice.

Quantitative PCR was performed using the DNA Engine with Chromo 4 Detector (MJ Research, Waltham, MA, USA). The following were added to a final reaction volume of 20 µl: 1x SuperMix (Platinum SYBR Green qPCR Kit; Invitrogen), cDNA (2 μl), and 0.5 µM of each primer. The amplification conditions were as follows: 50°C (2 min); 95°C (5 min); followed by 50 cycles of 95°C (15 s) and 60°C (30 s).

### 2.7 Western blot analyses

Protein was extracted from liver tissue in RIPA buffer containing phosphatase and protease inhibitors. 50-100mg liver tissue was added in 1ml RIPA buffer and homogenize using a homogenizer at 4°C; and then centrifuge the lysate at 12000 x g for 15 minutes at 4°C; collected the supernatant and kept on ice.

The concentrations of protein were determined by bicinchoninic acid (BCA) assay. The lysate was diluted 20-fold using PBS and mixed with work solution (prepared as according to the kit operating instructions), incubate at room temperature for one hour and measure the absorbance at 562 nm. Calculate the concentration of the protein to be measured from the standard curve.

Proteins in SDS-loading buffer were subjected to SDS-12% polyacrylamide gel electrophoresis (PAGE) and transferred to a PVDF membrane (Bio-Rad, Hercules, CA, USA). Antibodies against LC3B, Atg7, Atg5, Beclin-1, p62, β-actin (Cell Signaling Technology Inc., Santa Cruz, CA, USA), ASPP2 (Sigma, St. Louis, MO, USA) were used for western blot analysis. The membranes were incubated with primary antibodies (1:500-1000) diluted in 5% skim milk overnight at 4°C, washed with TBST buffer. Then, the membranes were incubated with the appropriate horseradish peroxidase-conjugated secondary antibody (1:2000) diluted in 5% skim milk for 1 hour at room temperature. SuperSignal West Pico chemiluminescent substrates (Thermo Fisher Scientific, Rockford, IL, USA) were used for chemiluminescence development. Finally, the gray values of the bands were examined by ImageJ to make comparisons between different groups.

### 2.8 Atg7 small interfering RNA treatment *in vivo*

Autophagy was inhibited with an siRNA against Atg7 (3 mg/kg; Jima, Suzhou); the sequence was 5′-GCAUCAUCUUCGAAGUGAATT-3′. Atg7 knockdown was achieved by siRNA using an Entranster™ *in vivo* transfection reagent (Engreen Biosystem Co, Beijing) via hydrodynamic tail vein injection in mice. Scrambled siRNA (3 mg/kg) was used as a control. The processes were performed according to the manufacturer’s instructions. To be detailed, diluted 25 μl of Entranster™ reagent with 25μl of 10% dextrose solution to a final volume of 50μl and mixed well; diluted 12.5 μg of nucleic acid to 1 μg/μl with DEPC water, added 12.5ul of water, then added 25 μl of 10% glucose solution (w/v) to a final volume of 50 μl and mixed thoroughly; added the diluted transfection reagent to the diluted nucleic acid solution at room temperature and mix with sufficient shaking and then incubated at room tempreture for 15min; the mixture injected into mice via tail vein.

### 2.9 Determination of hepatic caspase-3 activity

To examine the activity of caspase-3 in liver tissues, liver homogenates were prepared in lysis buffer and analyzed using a colorimetric caspase-3 assay kit (Chemicon International Co. USA) according to the manufacturer’s instructions. To be detailed, 10 mg of tissue was added in 100 µl lysate and homogenized on an ice; and then kept on ice for 5 min; centrifuge at 20,000g for 15 minutes at 4°C; and then transfer the supernatant to a pre-cooled centrifuge tube; mix the lysate with buffer and Ac-DEVD-*p*NA (2mM); incubated the mixture at 37°C for 60 min; finally, the absorbance can be measured to calculate caspase-3 activity.

### 2.10 Immunofluorescence staining

Paraffin sections were treated with xylene 3 times for 10 minutes each. The sections were hydrated through a graded alcohol series and then rinsed three times with distilled water. The slides were permeabilized in 0.01% Triton X-100, and then blocked in 10% goat serum in 3% bovine serum albumin (BSA, Sigma, St. Louis, MO, USA) for 60 minutes. Then, incubated with ASPP2 mouse polyclonal antibodies (1:200, Abcam, Cambridge, MA) diluted in 3% BSA overnight at 4°C. After washing with PBS three times, the slides were incubated with Alexa Fluor^®^ 568 goat anti-rabbit IgG (1:200, Invitrogen, Grand Island, NY) for 45 minutes at room temperature. After washing with PBS three times, the nuclei were stained with DAPI (1 μg/ml, Shizebio, Shanghai) for 10 min. Images were obtained on a Nikon Eclipse E800 fluorescence microscope.

### 2.11 Isolation and treatment of murine bone marrow-derived macrophages

Murine bone marrow-derived macrophages (BMMs) were isolated from bone marrow from 6- to 10-week-old BALB/c male mice. The detailed procedure is as follows: The anaesthetized mice were sterilized using 75% alcohol, followed by separating the bones of the limbs using sterile forceps and scissors, and then remove excess muscle tissue. The ends of the bones were cut off and a syringe was inserted from one end and DMEM was injected to flush out the bone marrow cells. The obtained cells were then centrifuged to remove impurities. The bone cells were cultured in conditioned medium (DMEM containing 10% fetal bovine serum, 1% penicillin/streptomycin and 20% L929) for 6 days to obtain macrophages. To identify the isolated BMMs, we performed CD68 (marker of macrophages) staining of the differentiated BMMs using immunofluorescence and the results showed that the purity of the isolated BMMs was significantly high (**Supplementary Figure 2**).

To prepare macrophage-conditioned media, BMMs from wild-type mice and ASPP2^+/-^ mice were seeded in six-well plates at a density of 1 × 10^6^ cells per well in 3 ml of complete medium. BMMs were treated with LPS (20 ng/ml) for 6 hours. The BMMs-conditioned media was collected, clarified by centrifugation at 400 × g and stored at -80°C until use.

### 2.12 siRNA transfection

To investigate the impact of IL-6 on TNF-α expression in BMMs, siRNA oligonucleotides against IL-6 (5 µM) and a negative control (5 µM) were transfected into ASPP2^+/-^ BMMs and wild-type BMMs. To investigate the impact of ASPP2 on the sensitivity of hepatocytes to apoptosis, siRNA against ASPP2 and (5 µM) and a negative control (5 µM) were transfected into primary hepatocytes. All siRNA oligonucleotides were purchased from GenePharma (Shanghai, China), and transfection was performed with Lipofectamine 2000 Reagent (Invitrogen, Thermo Fisher Scientific, Carlsbad, CA) according to the protocol. In detail, for the transfection mixture for one well of a 12-well plate with 70% confluent cells, 4.5 µl of Lipofectamine^®^ Reagent and 1 µg siRNA were diluted in 75 µl of Opti-MEM^®^ Medium. Then, the two dilutions were combined and incubated for 5 min at room temperature. Finally, a total volume of 125μl of transfection mixture was added dropwise to each well and incubated for 24-36h.

### 2.13 Isolation and treatment of primary mouse hepatocytes

6- to 10-week-old BALB/c male mice were used to isolate primary hepatocytes. As previously described (13), the liver was perfused with collagenase-containing Hanks’ solution; and the liver was excised from the abdominal cavity and transferred to a sterile 6 cm dish and shaken with sterile forceps; then the suspension was filtered using a 70 µm sieve; hepatocytes were isolated by Percoll density centrifugation, and then seeded in the collagen-coated six-well plates and incubated for 24-48 hours. BMMs-conditioned media (CM) were used to stimulate primary hepatocytes for 24 h.

### 2.14 LDH and CCK-8 assay

To investigate the cell death rate, cells were plated at a density of 1×10^4^ per well in a 96-well plate and treated as previously described. The cell death rate was performed with an LDH cytotoxicity assay kit (Beyotime, Shanghai, China) according to the manufacturer’s instructions, and cell viability was measured by cell counting kit-8 assay (Beyotime).

### 2.15 Flow cytometric analysis

An annexin V-PE/7-AAD double-staining assay (Nanjing Key Gen Biotech, Co., Nanjing, China) was used to quantify hepatocyte apoptosis by flow cytometric analysis. The experimental method was performed as previously described (14). The cells were washed with PBS buffer and resuspended in 100 µl of binding buffer containing 10 µl of PE and 7-AAD, incubate at room temperature in the dark. Then, the samples were analyzed by a Calibur flow cytometer (BD Bioscience, Franklin Lakes, NJ, USA), and the data were analyzed with FlowJo software. The cell apoptosis rate was determined by the sum of late-phase apoptotic cells (PE positive and 7-AAD positive) and early-phase apoptotic cells (PE positive and 7-AAD negative).

### 2.16 Statistical analyses

The results are shown as the mean ± SEM. Statistical analyses were performed using an unpaired Student’s t test, and p<0.05 (two tailed) was considered significant.

## 3 Results

### 3.1 ASPP2 is increased in the livers of ALI patients and mouse models induced by D-GalN/LPS

**Figure 1:**
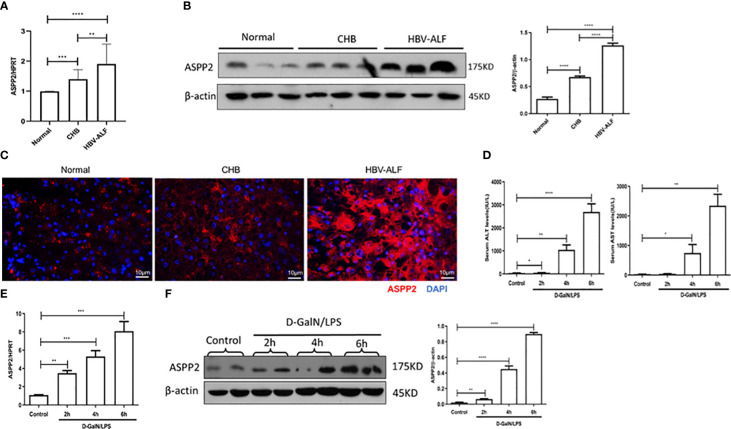
ASPP2 is increased in the livers of ALI patients and mouse models induced by D-GalN/LPS. **(A–C)** Liver tissue obtained from normal controls, patients with CHB and HBV-ALI were analyzed the expression level of ASPP2. **(A)** Gene expressions of ASPP2 was measured by qRT-PCR. **(B)** Protein expression levels of ASPP2 was measured by western blot assays. **(C)** The expression of ASPP2 was measured by immunofluorescence staining. **(D–F)** Mice were intraperitoneally injected with D-GalN (700 mg/kg) and LPS (10 μg/kg) at 2-, 4- and 6- hours (12 mice/group). The mice in the control group (n = 8) were injected with PBS only. **(D)** Serum AST and ALT enzyme levels. **(E)** Gene expressions of ASPP2 was measured by qRT-PCR. **(F)** Protein expression levels of ASPP2 was measured by western blot assays. *p<0.1, **p<0.01, ***p<0.001, ****p<0.0001.

To investigate whether ASPP2 is associated with the pathogenesis of ALI, we initially assessed the expression of ASPP2 in liver tissue from normal controls, patients with CHB and patients with HBV-ALI. The results suggested that the mRNA level of ASPP2 was increased in the liver tissue of patients with CHB and ALI compared with normal controls, and there was a significant increase in patients with ALI (**Figure 1A**). A similar change in the protein expression pattern of ASPP2 was observed in the liver tissues from normal controls, patients with CHB and ALI (**Figure 1B**). Moreover, liver sections from normal controls and patients with CHB and ALI were stained for ASPP2, the results indicated that ASPP2 was strong aggregation in the areas of hepatocyte injury, which was accompanied by nuclear fragmentation and disappearance in liver samples from patients with ALI (**Figure 1C**). Collectively, our results showed that the expression of ASPP2 was significantly enhanced in liver tissue from patients with ALI.

To further investigate the expression of ASPP2 in the development of acute liver injury, we induced ALI in mice with D-GalN/LPS at different time points. The levels of serum ALT and AST were analyzed, which gradually increased with prolonged drug treatment (**Figure 1D**). Importantly, the mRNA and protein levels of ASPP2 in the liver tissue of mice increased with the severity of liver injury (**Figures 1E, F**).

Altogether, our data demonstrated that the expression of ASPP2 was significantly increased in the liver tissue of ALI patients and mouse models.

### 3.2 Knockdown of ASPP2 protects against D-GalN/LPS-induced acute liver injury

**Figure 2:**
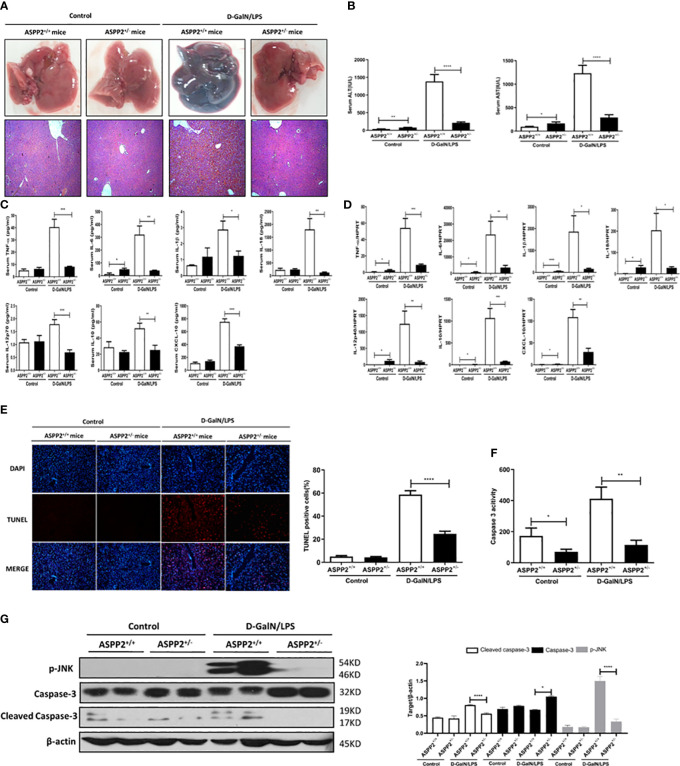
Knockdown of ASPP2 protects against D-GalN/LPS-induced acute liver injury. ASPP2^+/+^ mice and ASPP2^+/-^ mice were intraperitoneally injected with D-GalN/LPS for 6 hours (12 mice/group). **(A)** Representative livers and H&E staining of livers from different groups. **(B)** Serum AST and ALT enzyme levels from different groups. **(C)** Measurement of cytokine levels in the serum of mice in different groups by the Luminex Milliplex^®^ MAP Kit (TNF-α, IL-6, IL-1β, IL-18, IL-12, IL-10, CXCL-10, IL-13, IL-22). **(D)** Analysis of the gene expression levels of cytokines (TNF-α, IL-6, IL-1β, IL-18, IL-10, IL-12, CXCL-10) in liver tissues from different groups by qRT-PCR. **(E)** TUNEL staining (red) liver tissue at 6 h after D-GalN/LPS administration. Original magnification×200. **(F)** Caspase 3 activity of livers was measured from different groups. **(G)** Protein expression levels of p-JNK, caspase 3 and cleaved caspase 3 was measured by western blot assays. *p<0.1, **p<0.01, ***p<0.001, ****p<0.0001.

As the expression of ASPP2 was increased in patients and mouse models with ALI, we next explored its functional contribution to the development of acute liver injury. Acute liver injury was induced in ASPP2^+/-^ mice and ASPP2^+/+^ mice with D-GalN/LPS treatment for 6 hours. Histological examination of the livers of mice in the ASPP2^+/-^ group showed significantly attenuated liver injury according to the gross morphology, as well as a significantly decreased area of hepatocyte injury with loss of hepatic architecture and inflammatory cell infiltration around the blood vessels (**Figure 2A**). Consistently, ASPP2^+/−^ mice were significantly protected from D-GalN/LPS-induced liver injury, as indicated by decreased serum AST and ALT levels when compared with ASPP2^+/+^ mice (**Figure 2B**).

To determine the impact of ASPP2 knockdown on the induction of inflammatory cytokines by D-GalN/LPS-treated ALI, liquid phase microarray technology was used to examined the levels of cytokines in serum samples. The results showed that ASPP2 knockdown attenuates the serum level of cytokines including tumor necrosis factor a (TNF-α), interleukin-6 (IL-6), IL-1β, IL-18, IL-12p70, IL-10 and CXCL-10 (**Figure 2C**). Next, we further assessed hepatic mRNA expression of these inflammatory mediators in liver tissue by RT-PCR. Consistent with the above findings, the transcription levels of various cytokines were generally reduced in ASPP2^+/−^ mice compared with ASPP2^+/+^ mice during acute liver injury (**Figure 2D**).

Given the evidence that hepatocyte apoptosis is a key contributor to the pathogenesis of ALI, we next evaluated the impact of ASPP2 knockdown on hepatocyte apoptosis. To evaluate hepatocyte apoptosis in liver tissue induced by D-GalN/LPS, TUNEL staining was performed with liver tissue sections. The results indicated that TUNEL-positive cells were significantly reduced in ASPP2^+/−^ mice compared with ASPP2^+/+^ mice (**Figure 2E**). Moreover, caspase-3 activity in liver tissue was measured, and the results showed a significant reduction in the livers of ASPP2^+/−^ mice compared with the ASPP2^+/+^ mice after treatment with D-GalN/LPS (**Figure 2F**). Furthermore, the protein level of p-JNK and cleaved caspase-3 significantly decreased in the livers of ASPP2^+/−^ mice compared with the ASPP2^+/+^ controls (**Figure 2G**). These results indicated that ASPP2 knockdown significantly reduced hepatocyte apoptosis during ALI induced by D-GalN/LPS.

In summary, knockdown of ASPP2 in mice attenuated liver injury through inhibition of the excessive activation of inflammatory response and decreasing hepatocyte apoptosis in the liver induced by D-GalN/LPS.

### 3.3 Knockdown of ASPP2 reduced hepatocyte apoptosis through autophagy activation during ALI

**Figure 3:**
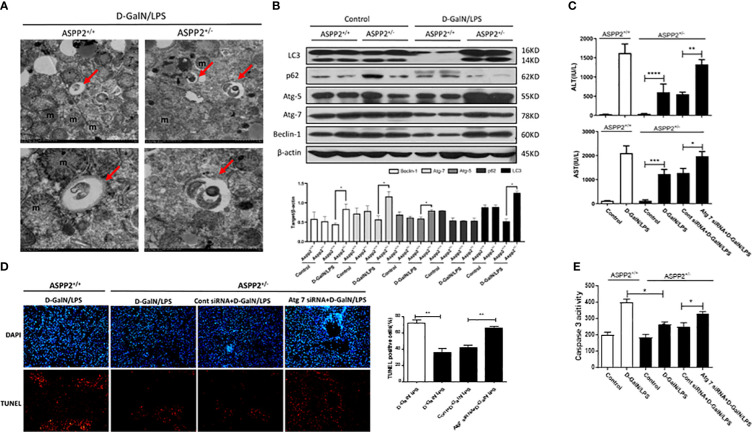
Knockdown of ASPP2 reduced hepatocyte apoptosis through autophagy activation during ALI. **(A, B)** ASPP2^+/+^ mice and ASPP2^+/-^ mice were intraperitoneally injected with D-GalN/LPS for 6 hours (12 mice/group). **(A)** Autophagosomes was observed by electron microscope in the livers from different groups. **(B)** Protein expression levels of LC3, p62, ATG-5, Atg-7 and Beclin-1 were measured by western blotting. **(C–E)** Mice were pretreated with Atg7 or negative control siRNA (3mg/kg, iv) for 48 hours prior to D-GalN/LPS exposure (n = 12). **(C)** Serum AST and ALT enzyme levels from different groups. **(D)** TUNEL staining (red) liver tissue from different groups. Original magnification×200. **(E)** Caspase 3 activity of livers was measured from different groups. *p<0.1, **p<0.01, ***p<0.001, ****p<0.0001.

Autophagy is a lysosomal-dependent self-degradation pathway that maintains liver homeostasis through its role in energy balance and in the quality control of the cytoplasm by removing misfolded proteins, damaged organelles, and lipid droplets. The previous study has shown that downregulation of ASPP2 facilitates autophagic flux in hepatocellular carcinoma cells. To investigate the impact of ASPP2 deficiency on the activation of autophagy during the process of acute liver injury, we initially examined the formation of autophagosomes with transmission electron microscope (TEM) in the livers of ASPP2^+/−^ and ASPP2^+/+^ mice induced by D-GalN/LPS. The results revealed an increase of autophagosomes in liver tissue of ASPP2^+/−^ mice but not in ASPP2^+/+^ mice treated with D-GalN/LPS (**Figure 3A**). In line with the results of the TEM analyses, knockdown of ASPP2 increased the expression of autophagy-related proteins, including LC3, Atg5, Atg7 and Beclin-1, and decreased the level of p62 in liver tissues induced by D-GalN/LPS (**Figure 3B**). These data demonstrated that ASPP2 deficiency promoted autophagy activation in the liver during ALI.

Furthermore, we hypothesized that knockdown of ASPP2 may protect against liver injury induced by D-GalN/LPS through activation of autophagy. Atg7 is a key molecule that contributes to the formation of autophagosomes, which was knockdown by specific siRNAs to inhibit autophagy in ASPP2^+/−^ mice. Mice liver injury was evaluated by the levels of serum AST and ALT, and the results indicated that knockdown of Atg7 in ASPP2^+/−^ mice abolished the protective effect of ASPP2 deficiency during ALI induced by D-GalN/LPS (**Figure 3C**). Furthermore, we stained liver sections with TUNEL assay, the results showed that TUNEL-positive hepatocytes were significantly increased in the livers of Atg7-siRNA-treated ASPP2^+/−^ mice (**Figure 3D**). In agreement with these data, we observed a strong increase in caspase3 activity in the liver samples of ASPP2^+/−^ mice that were treated with Atg7-siRNA (**Figure 3E**). Therefore, these results demonstrated that the hepatoprotective mechanisms of ASPP2 knockdown in a manner dependent on activation of the autophagy pathway.

### 3.4 ASPP2-depleted BMMs protect hepatocytes from apoptosis by reducing TNF-α levels *in vitro*

**Figure 4:**
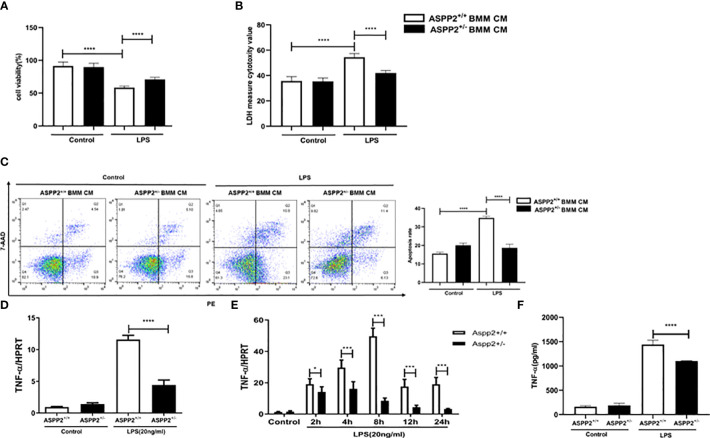
ASPP2-depleted BMMs protect hepatocytes from apoptosis by reducing TNF-α levels *in vitro.* **(A, B)** Primary hepatocytes were treated with conditioned medium (CM) of ASPP2^+/+^ BMMs or ASPP2^+/-^ BMMs that with or without LPS (20ng/ml). **(A, B)** The cell viability and cell death rate of primary hepatocytes were measured by CCK-8 assays and LDH assays, respectively. **(C)** The cell apoptosis rate of primary hepatocytes was measured by flow cytometry. **(D)** ASPP2^+/+^ BMMs or ASPP2^+/-^ BMMs were treated with LPS (20ng/ml) for 6 hours, the level of TNF-α was analyzed by qRT-PCR. **(E)** ASPP2^+/+^ BMMs or ASPP2^+/-^ BMMs were treated with LPS (20ng/ml) as the indicated time points, the level of TNF-α was analyzed by qRT-PCR. **(F)** ASPP2^+/+^ BMMs or ASPP2^+/-^ BMMs were treated with LPS (20ng/ml) for 6 hours, the level of TNF-α in medium was analyzed by ELISA. *p<0.1, ***p<0.001, ****p<0.0001.

We demonstrated that knockdown of ASPP2 regulates the hepatic inflammatory response in acute liver injury induced by D-GalN/LPS. To further explore the regulatory effect of ASPP2 on the expression of cytokines, we isolated BMMs from the bone marrow of WT mice and ASPP2^+/−^ mice and treated them with LPS, a component of gram-negative bacteria. Then, the conditioned medium (CM) of BMMs was collected and incubated with primary hepatocytes from WT mice for 24 h to analyze cell death rate and the apoptosis rate. The results showed that cell death rate of primary hepatocytes was significantly decreased when treated with ASPP2^+/−^ BMM-CM (**Figures 4A, B**), and the cell apoptosis rate was significantly reduced in hepatocytes incubated with ASPP2^+/−^ BMM-CM (**Figure 4C**). These results showed that the ASPP2-deficient BMM-CM could significantly protect hepatocytes from apoptosis.

According to previous studies, TNF-α is a potent cytokine that exerts pleiotropic inflammatory and immunological functions, and its circulating level are increased in ALI patients and are associated with a poor prognosis. Next, we sought to clarify whether the protective effect of ASPP2^+/-^ BMM-CM on hepatocytes was mediated by TNF-α that released into medium. The transcriptional level of TNF-α was assessed by RT-PCR, and the results showed that knockdown of ASPP2 significantly decreased the expression level of TNF-α in BMMs stimulated by LPS (**Figure 4D**). Specifically, the expression profile of TNF-α was measured in BMMs treated with LPS at different time points, which showed that ASPP2 deficiency significantly decreased the expression of TNF-α (**Figure 4E**). Consistently, secreted TNF-α levels from LPS-treated BMMs was measured, the results showed the concentration of TNF-α significantly reduction in ASPP2^+/−^ BMMs compared with ASPP2^+/+^ BMMs when treated with LPS (**Figure 4F**).

Altogether, these results indicated ASPP2 deficiency in BMMs reduced the LPS-triggered expression of TNF-α, which protects hepatocytes from apoptosis *in vitro*.

### 3.5 ASPP2-depleted BMMs decreased TNF-α by up-regulation of IL-6

**Figure 5:**
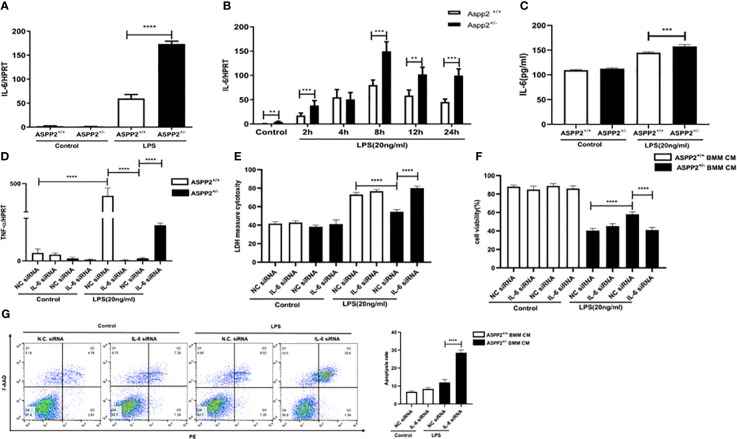
ASPP2-depleted BMMs decreased TNF-α by up-regulation of IL-6. **(A)** ASPP2^+/+^ BMMs or ASPP2^+/-^ BMMs were treated with LPS (20ng/ml) for 6 hours, the level of IL-6 was analyzed by qRT-PCR. E. ASPP2^+/+^ BMMs or ASPP2^+/-^ BMMs were treated with LPS (20ng/ml) as the indicated time points, the level of IL-6 was analyzed by qRT-PCR. E. ASPP2^+/+^ BMMs or ASPP2^+/-^ BMMs were treated with LPS (20ng/ml) for 6 hours, the level of IL-6 in medium was analyzed by ELISA. **(D)** IL-6 siRNA or negative control siRNA was transected into ASPP2^+/+^ BMMs and ASPP2^+/-^ BMMs that with or without LPS (20ng/ml) stimulation. The level of TNF-α in BMMs were analyzed by qRT-PCR. **(E, F)** primary hepatocytes were treated with conditioned medium (CM) collected from **(D)**. The cell viability and cell death rate of primary hepatocytes were measured by CCK-8 assays and LDH assays, respectively. **(G)** Primary hepatocytes were treated with CM the same as **(D)**. The cell apoptosis rate of primary hepatocytes was measured by flow cytometry. *p<0.1, **p<0.01, ***p<0.001, ****p<0.0001.

Previous study reported that IL-6 protects against liver injury induced by LPS by regulating proinflammatory cytokines and chemokines (15). To determine the impact of ASPP2 deficiency on the expression of IL-6 in BMMs, the transcriptional level of IL-6 was assessed by RT-PCR, and the results showed that knockdown of ASPP2 significantly increased the expression of IL-6 in BMMs stimulated by LPS (**Figure 5A**). Moreover, the expression profile of IL-6 was significantly increased in BMMs that treated with LPS at different time points (**Figure 5B**). Consistently, secreted IL-6 levels from LPS-treated BMMs showed IL-6 level significantly increased in ASPP2^+/−^ BMMs compared with ASPP2^+/+^ BMMs when treated with LPS (**Figure 5C**). These results showed that knockdown of ASPP2 promoted IL-6 expression in BMMs when treated with LPS. Next, to explore whether up-regulated IL-6 inhibited the expression of TNF-α, specific siRNA was used to inhibited IL-6 expression in ASPP2^+/-^ BMMs. The results showed that knockdown of IL-6 significantly increased the expression of TNF-α in ASPP2^+/−^ BMMs treated with LPS (**Figure 5D**).

To explored the ASPP2^+/−^ BMM-CM that IL-6 knockdown weather contributes to hepatocyte apoptosis. The medium from ASPP2^+/−^ BMMs that treated with IL-6 siRNA showed pro-injury effect on hepatocytes, which significantly decreased cell viability and increased cell death rate, abolished the protective effect of ASPP2^+/−^ BMMs supernatants on hepatocytes (**Figures 5E–G**).

Collectively, our results demonstrated that ASPP2^+/−^ BMMs decreased the expression of TNF-α through up-regulating of IL-6. However, the level of IL-6 was decreased in ASPP2^+/−^ murine models with ALI but increased in ASPP2^+/−^ BMMs treated with LPS, which indicates a more complex regulatory network exists *in vivo*.

### 3.6 ASPP2^+/−^ BMMs promote IL-6 expression through autophagy induction

**Figure 6:**
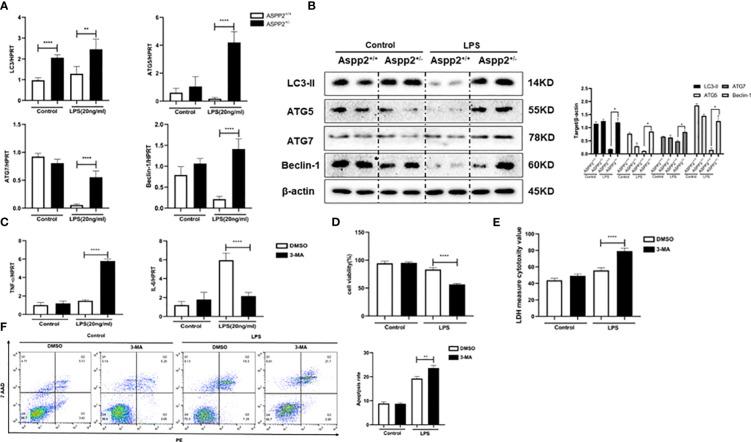
ASPP2^+/−^ BMMs promote IL-6 expression through autophagy induction. **(A, B)** ASPP2^+/+^ BMMs or ASPP2^+/-^ BMMs were treated with LPS (20ng/ml) for 12 hours. **(A)** The gene expression levels of autophagy related genes (LC3, ATG-7, ATG-5, Beclin-1) were analyzed by qRT-PCR. **(B)** The protein expression levels of autophagy related genes (LC3, ATG-7, ATG-5, Beclin-1) were analyzed by western blotting. **(C)** DMSO and 3-MA were pretreated with cells 2 hours before LPS stimulation. The gene expression levels of cytokines (IL-6, TNF-α) in cells were analyzed by qRT-PCR. **(D, E)** Primary hepatocytes were incubated with conditioned medium (CM) of BMMs treated as **(C)** The cell viability and cell death rate of primary hepatocytes were measured by CCK-8 assays and LDH assays, respectively. **(F)** The cell apoptosis rate of primary hepatocytes was measured by flow cytometry. *p<0.1, **p<0.01, ****p<0.0001.

We showed that ASPP2 deficiency promoted autophagy activation in ALI mice, which could reduce hepatocyte apoptosis and ameliorate liver injury. Therefore, we analyzed the expression of autophagy markers in ASPP2^+/−^ BMMs. The results showed that autophagy markers, such as LC3, ATG5, ATG7, and Beclin-1, were significantly increased in ASPP2^+/−^ BMMs compared with ASPP2^+/+^ BMMs when treated with LPS (**Figures 6A, B**).

We next explored whether the activation of autophagy in ASPP2^+/−^ BMMs impact the expression of IL-6 and TNF-α. 3-Methyladenine (3-MA) was used to inhibit autophagy in ASPP2^+/−^ BMMs, and we measured the RNA levels of TNF-α and IL-6. The results showed that inhibition of autophagy in ASPP2^+/−^ BMMs significantly decreased the level of IL-6, and increased the level of TNF-α (**Figure 6C**). Next, to investigate whether activation of autophagy in ASPP2^+/-^ BMMs affects their protective effect on hepatocytes, the supernatant of BMMs was collected and incubated with primary hepatocytes. We observed that the viability of hepatocytes decreased significantly and the cell apoptosis rate increased significantly when autophagy was inhibited in ASPP2^+/−^ BMMs by 3-MA (**Figures 6D–F**). These results showed that autophagy induction in ASPP2^+/−^ BMMs significantly reduced primary hepatocyte apoptosis through upregulation of IL-6 to inhibited TNF-α.

### 3.7 Upregulation of ASPP2 sensitizes hepatocytes to TNF-α-induced apoptosis

**Figure 7:**
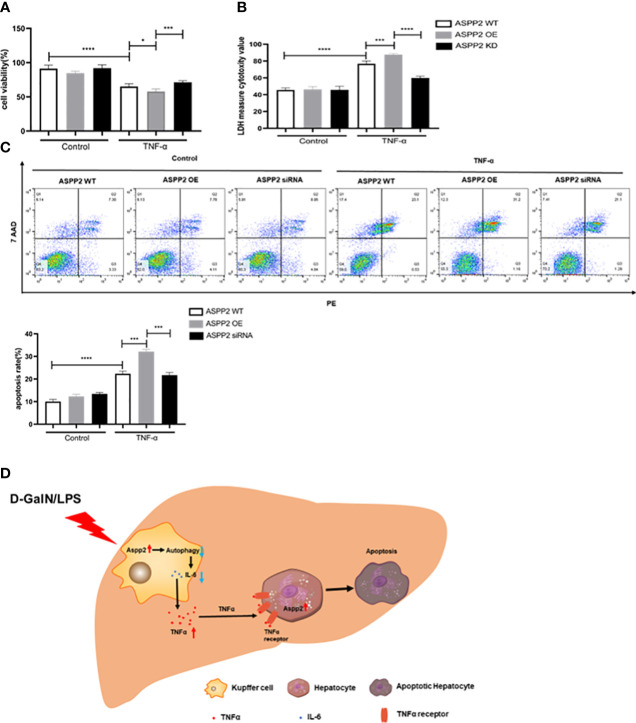
Upregulation of ASPP2 sensitizes hepatocytes to TNF-α-induced apoptosis. **(A-C)** Primary hepatocytes were transfected with ASPP2 over-expression (OE) plasmid or ASPP2 siRNA, and treated with TNF-α for 12 hours. **(A, B)** The cell viability and cell death rate of primary hepatocytes were measured by CCK-8 assays and LDH assays, respectively. **(C)** The cell apoptosis rate of primary hepatocytes was measured by flow cytometry. **(D)** The graphic abstract of this study. In ALI mice induced by D-GalN/LPS, ASPP2 was increased in liver Kupffer cells, which suppressed the level of IL-6 and increased the level of TNF-α through inhibition autophagy to promote hepatocytes apoptosis. In addition, upregulation of ASPP2 in hepatocytes promoted cell apoptosis induced by TNF-α. *p<0.1, ***p<0.001, ****p<0.0001.

We determined ASPP2 aggravates liver injury through promoting the release of TNF-α from Kupffer cell in ALI mouse. However, whether up-regulation of ASPP2 in hepatocytes affects cell apoptosis induced by TNF-α remains unclear. Primary hepatocytes were isolated from WT mice, which transfected with over-expressed (OE) plasmid or siRNA to regulate the expression of ASPP2, and then treated with TNF-α. The results indicated ASPP2-OE hepatocytes showed decreased cell viability, and increased cell death rate compared with WT hepatocytes upon stimulation with TNF-α (**Figures 7A, B**). Moreover, knockdown of ASPP2 in hepatocytes showed increased cell viability when treated with TNF-α. Consistently, the apoptosis rate showed a significant increase in ASPP2-OE hepatocytes but decreased in ASPP2-knockdown hepatocytes when treated with TNF-α (**Figure 7C**). These results indicated that ASPP2 sensitizes hepatocytes to TNF-α-induced apoptosis.

## 4 Discussion

ALI caused by HBV is very common in China, with an extremely poor prognosis and high mortality. In the present study, we demonstrated that the expression of ASPP2 was significantly increased in patients with ALI and in D-GalN/LPS-induced ALI mice. Furthermore, ASPP2 deficiency significantly ameliorated D-GalN/LPS-induced acute liver injury by reducing liver inflammation and hepatocyte apoptosis. Mechanically, knockdown of ASPP2 promoted the activation of autophagy signaling in the livers of ALI mice, and inhibition of the autophagy pathway counteracted the protective effect of ASPP2 deficiency on D-GalN/LPS-induced liver injury in mice. On one hand, ASPP2^+/-^ BMMs specifically upregulated the expression of IL-6 and suppressed the expression of TNF-α through autophagy activation, thus play a protective role to reduce hepatocyte apoptosis *in vitro*, on the other hand, ASPP2 up-regulated in hepatocytes promoted cell apoptosis induced by TNF-α (**Figure 7D**). Altogether, this study revealed that enhancing ASPP2 in liver magnified the inflammation response to promote liver injury by inhibiting autophagy during the process of ALI.

Although ASPP2, which is a binding partner of p53, has been extensively studied in the field of tumor suppression (16), our study highlights the critical role of ASPP2 in the progression of ALI. According to several studies, ASPP2 deficiency significantly alleviates the inflammatory response and reduces hepatocyte apoptosis during mouse acute liver injury induced by carbon tetrachloride (CCl_4_) (17), and ASPP2 plays an important role in hepatocyte apoptosis induced by TNF-α (18). Consistent with previous research, we showed that the expression of ASPP2 was significantly increased in ALI patients and D-GalN/LPS-induced ALI mice, and proved the role of ASPP2 in aggravating liver injury by promoting liver inflammation and inducing hepatocyte apoptosis in the progression of ALI.

In addition, we observed that ASPP2 deficiency significantly reduced the levels of several cytokines in the serum and liver tissue of mice during acute liver injury induced by D-GalN/LPS. Studies have shown that cytoplasmic ASPP2 regulates the NF-κB pathway by binding to IκB in the cytoplasm, which participates in the regulation of inflammatory responses during tumor development (10). A transcriptomic study showed that ASPP2-knockdown mice had reduced inflammatory responses to chronic HBV infection compared with control mice (19). In addition, ASPP2-knockdown mice exhibited reduced inflammatory responses during acute kidney injury caused by ischemia reperfusion (20). In this study, ASPP2 played a critical role in regulating inflammation, which deficiency resulted in the downregulation of several cytokines in liver tissue and serum. Furthermore, we demonstrated that enhancing ASPP2 promoted the expression of TNF-α by inhibiting IL-6 *in vitro*. Accumulating evidence has shown that IL-6 has significant functions in the regulation of the immune system (21), and IL-6-deficient mice exhibited a significant increase in TNF-alpha levels after liver injury in a recent study (22). Our findings indicated that ASPP2 deficiency in BMMs significantly decreases the level of TNF-α by upregulating IL-6.

Although ASPP2 has been proven to promote cell apoptosis in p53-independent manner in cancer cells, but the detailed mechanisms need to be explored. In this study, we found that ASPP2 depletion enhanced autophagic activity in hepatocytes to inhibit cell apoptosis *in vivo* and *in vitro*. However, inhibiting autophagy reversed the hepatoprotective effect of ASPP2 deficiency in hepatocytes. Researchers have reported that ASPP2 is a key regulator of BECN1-dependent autophagy and that downregulation of ASPP2 facilitates autophagic flux; ASPP2 inhibits autophagy by competing with ATG16 to bind ATG5/ATG12 to prevent ATG16/ATG5/ATG12 formation (23, 24). In addition, recent studies have proven that knockdown of ASPP2 could decrease TNF-α-induced hepatocyte apoptosis (18). Our findings proposed the new role of ASPP2 that inhibition of autophagy to promote cell apoptosis in hepatocytes, which accelerates disease progression during ALI.

It is now established that a large amount of hepatotoxic cytokine aggravates liver injury by promoting inflammatory response in the liver (4). In our study, we showed that ASPP2 promotes TNFα secretion in macrophages by inhibiting autophagy, thereby exacerbating liver inflammation. On the other hand, we investigated that upregulation of ASPP2 in hepatocytes makes them more sensitive to TNFα-induced hepatocyte apoptosis. During the progression of ALI, upregulated ASPP2 played an important role in both inflammatory cells and hepatocytes to exacerbate liver injury, which suggesting an important role of ASPP2 in ALI. The present study suggests that inhibition of ASPP2 would significantly reduce disease progression and improve prognosis in ALI patients, but the chemical inhibitors are needing further exploration.

However, there are some disadvantages in this study that need to be improved in the further work: (1) the upstream pathways causing ASPP2 upregulation in the D-GalN/LPS-induced ALI mouse model need to be further studied; (2) to clarify the role of ASPP2 in liver parenchymal and non-parenchymal cells, hepatocyte-specific ASPP2 knockout mice and macrophage-specific ASPP2 knockout mice should be used in the next study; (3) the detailed mechanism by which upregulated ASPP2 in hepatocytes promotes TNF-induced apoptosis needs to be further explored.

In summary, this study adds to the general understanding of mechanisms of ALI and provides new insight into the importance of ASPP2 in regulating liver injury. ASPP2 inhibition may provide not only the direct hepatocyte-protection that against cell apoptosis, but also exert immune modulation to reduce local inflammation. Further preclinical studies with ASPP2 inhibitors are warranted to pave the way for the development of a clinically applicable therapeutic strategy against liver failure.

## Data availability statement

The original contributions presented in the study are included in the article/**supplementary materials**, further inquiries can be directed to the corresponding authors.

## Ethics statement

The studies involving humans were approved by Medical Ethics Committee of Beijing YouAn Hospital. The studies were conducted in accordance with the local legislation and institutional requirements. The participants provided their written informed consent to participate in this study. The animal study was approved by Ethical Committee of Capital Medical University. The study was conducted in accordance with the local legislation and institutional requirements.

## Author contributions

XZ: Conceptualization, Methodology, Writing – review & editing. LX: Formal analysis, Investigation, Methodology, Writing – original draft. ZF: Formal analysis, Methodology, Writing – original draft. YG: Software, Supervision, Validation, Writing – review & editing. YT: Formal analysis, Investigation, Methodology, Writing – review & editing. YC: Formal analysis, Methodology, Writing – review & editing. DC: Conceptualization, Investigation, Resources, Writing – review & editing. FR: Conceptualization, Data curation, Funding acquisition, Resources, Writing – review & editing.
