# Supplementary material for: Enhancing ASPP2 promotes acute liver injury via an inflammatory immunoregulatory mechanism
Source: Front Immunol. 2024 May 22;15:1381735. doi: 10.3389/fimmu.2024.1381735 (PMC11150554; doi:10.3389/fimmu.2024.1381735)
Supplement: Supplementary file 1 [file DataSheet_1.docx]

Supplementary Materials

1. **Supplemental Figure**
   1. **Supplemental figure 1**


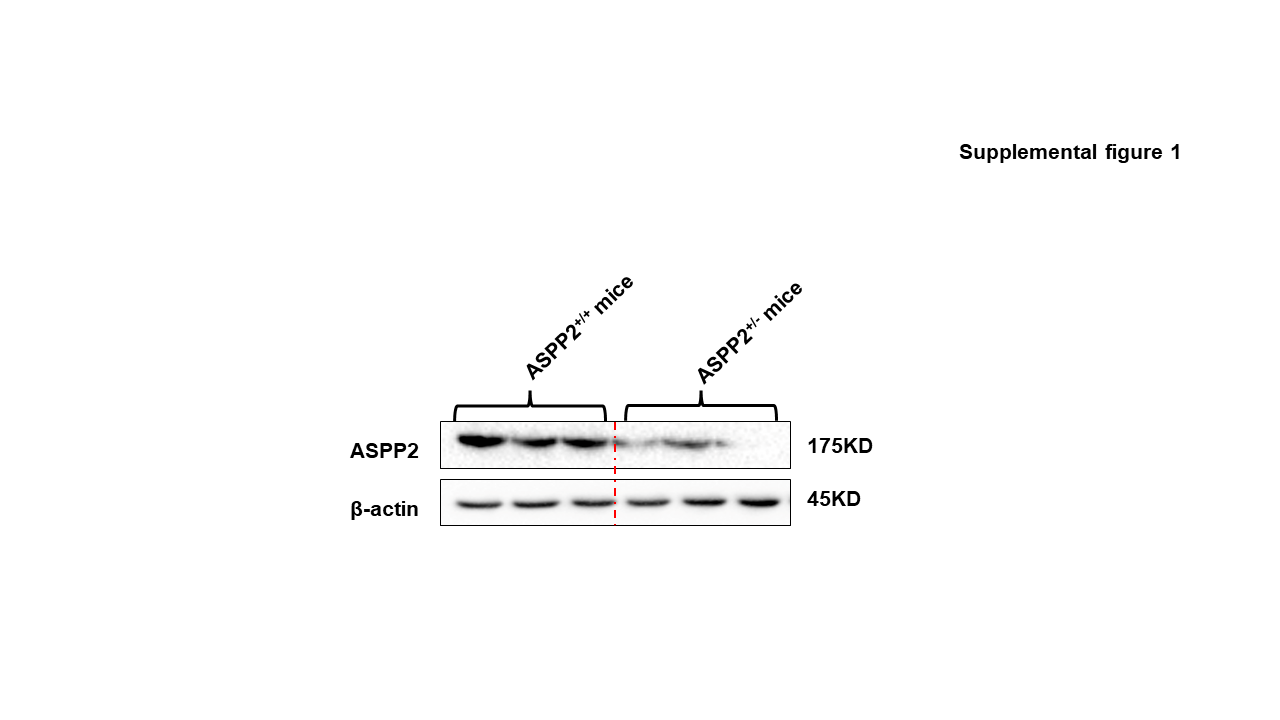


The expression of ASPP2 in liver tissue from ASPP2^+/-^ mice and ASPP2^+/+^ mice.

**1.2 Supplemental figure 2**


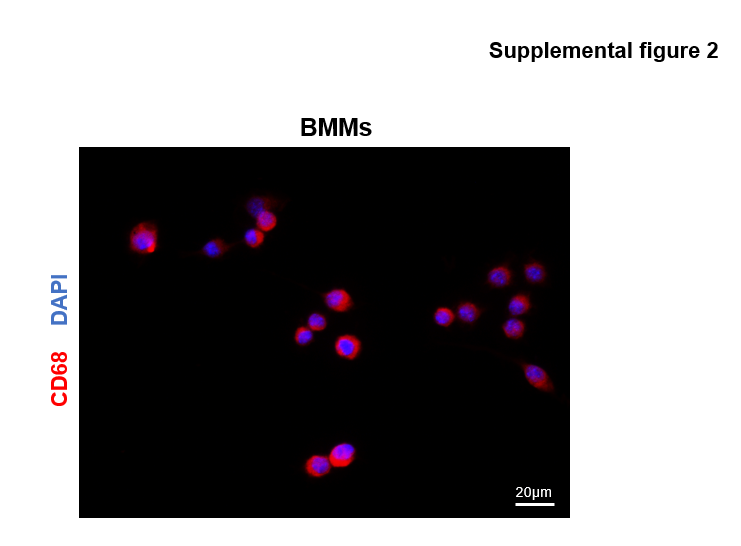


The isolated BMMs were stained with CD68 (marker of macrophages) and observed using immunofluorescence assay.

**1.3 Supplemental figure 3**


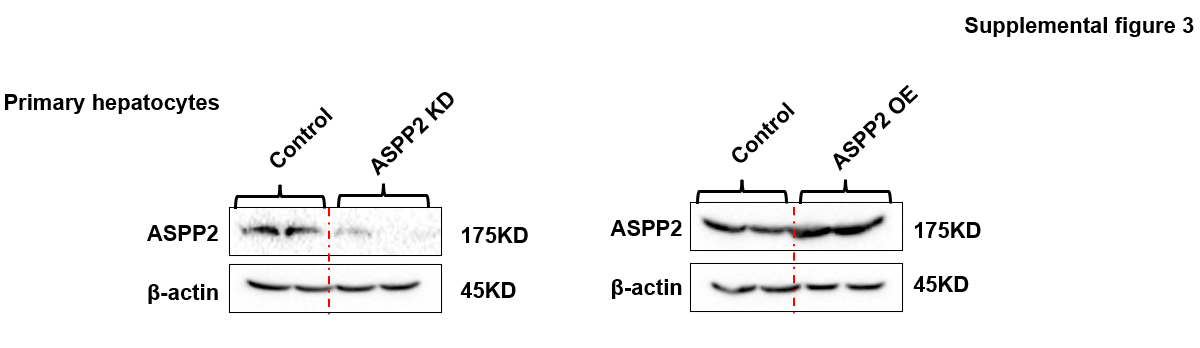


The expression of ASPP2 in primary hepatocytes treated with ASPP2 siRNA or ASPP2 over-expressed (OE) plasmid.

1. **Supplemental Table**

**Supplemental Table 1** General clinical characteristics of the study groups

| Parameters | Normal subjects (n=10) | CHB subjects (n=14) | HBV-ALF subjects (n=19) | P value |
| --- | --- | --- | --- | --- |
| Age (years) | 46.4±8.8 | 46.5±9.0 | 46.8±8.3 | >0.05 |
| Gender (Male/Female) | (4/6) | (6/8) | (12/7) | >0.05 |
| Alanine aminotransferase (U/L) | 26.3±5.3 | 62.9±14.0 | 1311±461.1 | <0.0001 |
| Aspartate aminotransferase (U/L) | 23.7±7.3 | 61.0±17.4 | 1364.0±555.9 | <0.0001 |
| Serum bilirubin (μmol/L) | 5.7±1.7 | 23.8±4.9 | 217.1±37.8 | <0.0001 |
| Prothrombin time (s) | 11.8±0.7 | 12.7±0.8 | 23.1±2.2 | <0.01 |
| HBsAg | - | Positive (n=14) | Positive (n=19) | - |
